# Supplementary material for: Inhibition of Plasmid Conjugation in Escherichia coli by Targeting rbsB Gene Using CRISPRi System
Source: Int J Mol Sci. 2023 Jun 24;24(13):10585. doi: 10.3390/ijms241310585 (PMC10342112; doi:10.3390/ijms241310585)

Supplementary data

Table S1. Oligonucleotides and primers used in this study.

Primers	Sequences (5'-3')	Vectors
**Oligonucleotides used for sgRNA synthesis**		
BspQI-B1-F	aaaGGTTTCCGCTGTTGCGCTAA	plv-dCas9-B1
BspQI-B1-R	aacTTAGCGCAACAGCGGAAACC	
BspQI-B2-F	aaaCCGTCAGTGCGAATGCGATG	plv-dCas9-B2
BspQI-B2-R	aacCATCGCATTCGCACTGACGG	
BspQI-B3-F	aaaTCGCGCTGGTGGTCTCCACG	plv-dCas9-B3
BspQI-B3-R	aacCGTGGAGACCACCAGCGCGA	
**Primers used for colony PCR**		
BspQI-B1-F	aaaGGTTTCCGCTGTTGCGCTAA	plv-dCas9-B1
sgRNA-R	GCGGAATATATCCCTAGGCCTGCAG	
BspQI-B2-F	aaaCCGTCAGTGCGAATGCGATG	plv-dCas9-B2
sgRNA-R	GCGGAATATATCCCTAGGCCTGCAG	
BspQI-B3-F	aaaTCGCGCTGGTGGTCTCCACG	plv-dCas9-B3
sgRNA-R	GCGGAATATATCCCTAGGCCTGCAG	
RP4-traG-F	AAAGCGGACAGCATCAGTAACGAA	
RP4-traG-R	GAGCTTGGTGGCCGCATAGTGTAG	
**Primers used for DNA sequencing**		
sgRNA-F	TGTTTGTCGGTGAACGCTCTCTACTAG	
sgRNA-R	GCGGAATATATCCCTAGGCCTGCAG	
**Primers used for RT-qPCR**		
16SrRNA-F	CCTACGGGAGGCAGCAG	
16SrRNA-R	ATTACCGCGGCTGCTGG	
rbsB-F	ATCGGATGTGATGGTCGTCG	
rbsB-R	AGTCGCTGCTAGTTTGCCAT	
traF-F	GGCAACCTCGTCGCCTTTA	
traF-F	GCAAGTCGGCGTGTTTTCG	
traJ-F	GCCCGTGATTTTGTAGCCC	
traJ-R	TGAAACCAAGCCAACCAGGAA	
trfAp-F	GAAGCCCATCGCCGTCGCCTGTAG	
trfAp-R	GCCGACGATGACGAACTGGTGTGG	
trfBp-F	CGCGGTCGCCATCTTCACG	
trfBp-R	TGCCCGAGCCAGTACCGCCAATG	
korA-F	TCGGGCAAGTTCTTGTCC	
korA-R	GCAGCAGACCATCGAGATA	
korB-F	CTGGTCGGCTTCGTTGTA	
korB-R	TGAAGTCACCCATTTCGGT	
trbA-F	TGGAAACTCCCCTACCTCTT	
trbA-R	CCACACTGATGCGTTCGTAT	
luxS-F	TGCGTGCCGAACAAAGAA	

luxS-R	CAGCCCATTGGCGAGATA
rbsD-F	AAGTCAGGCGGTAATTCGCA
rbsD-R	TTCCATGACGGCCTCAGAAC
rbsA-F	CTACCCGTGGCGTAGATGTC
rbsA-R	TGCGATCGCTCATGCCTAAT
rbsC-F	CAGCAAACCTCAGTGAACGC
rbsC-R	CAGCGCCAACAGAGAACCTA
rbsK-F	CGTCATCGACAGATGAACGTG
rbsK-R	CATAAAATGCGCCACCGTGT

Figure S1. Effect of CRISPRi system with sgRNAs (B1) on genes in *rbsDACBK* operon. ***, $p < 0.001$

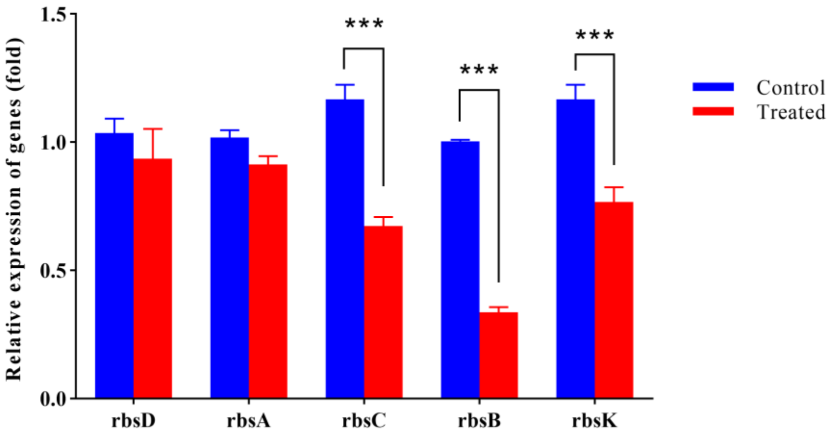

Supplement: Supplementary file 1 [file ijms-24-10585-s001.zip › ijms-2432708-supplementary.pdf]
